# Supplementary material for: Global Down-regulation of Gene Expression Induced by Mouse Mammary Tumor Virus (MMTV) in Normal Mammary Epithelial Cells
Source: Viruses. 2023 May 2;15(5):1110. doi: 10.3390/v15051110 (PMC10223558; doi:10.3390/v15051110)
Supplement: Supplementary file 1 [file viruses-15-01110-s001.zip › Supplementary Figures S1-S4.pdf]

viruses

MDPI

Supplementary Figures S1-S4

Global Downregulation of Gene Expression Induced by Mouse Mammary Tumor Virus (MMTV) in Normal Mammary Epithelial Cells

Waqar Ahmad[1¥], Neena G. Panicker[1¥], Shaima Akhlaq[1], Bushra Gull[1], Jasmin Baby[1], Thanumol A. Khader[1], Tahir A. Rizvi[2,3,4] and Farah Mustafa[1,3*]

Figure S1

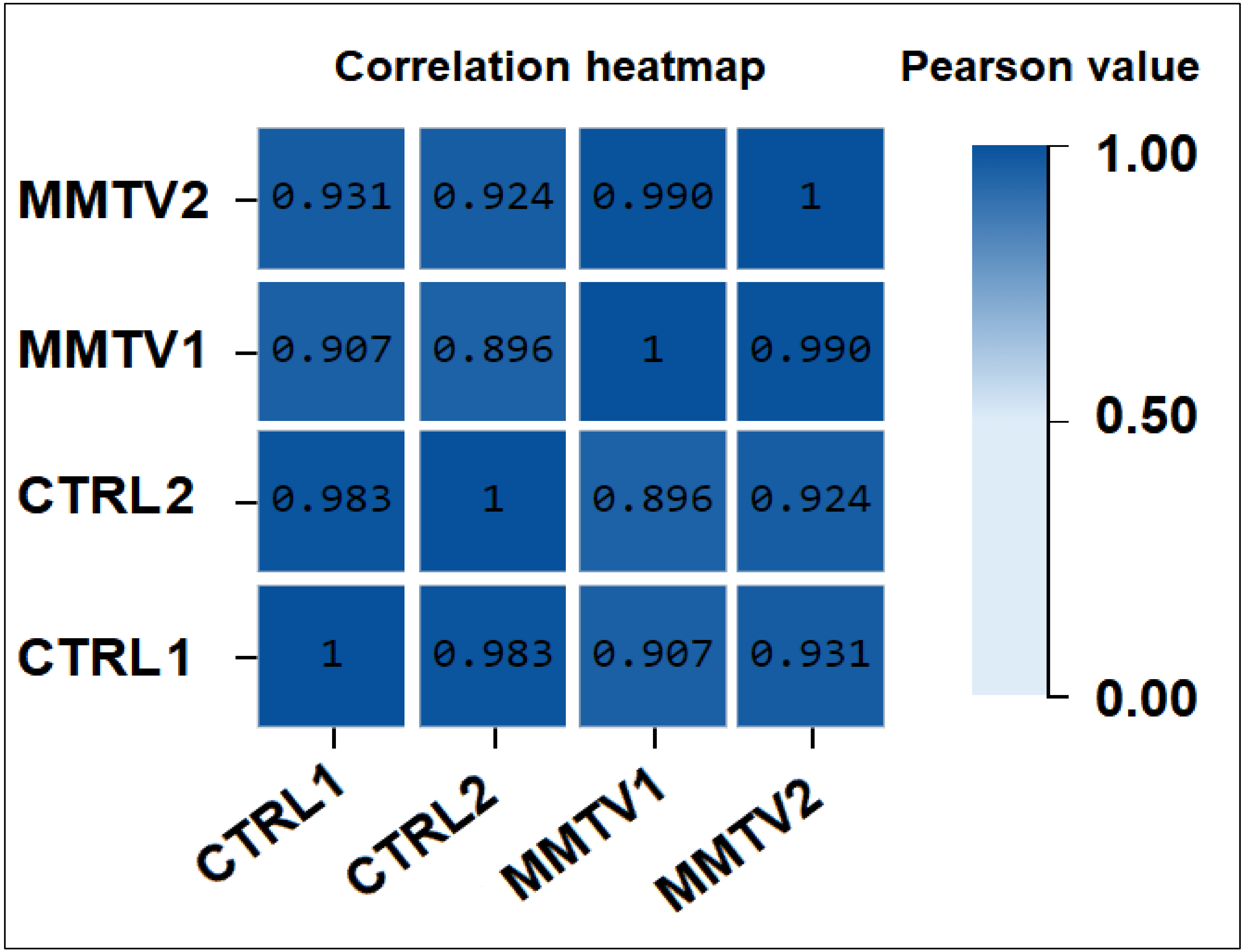

Figure S1: Pearson correlation graph of four samples sequenced in this study. The X and Y axes represent each sample. Color represents the correlation coefficient (darker colors represent higher correlations whereas lighter colors represent lower correlations.)

Figure S2

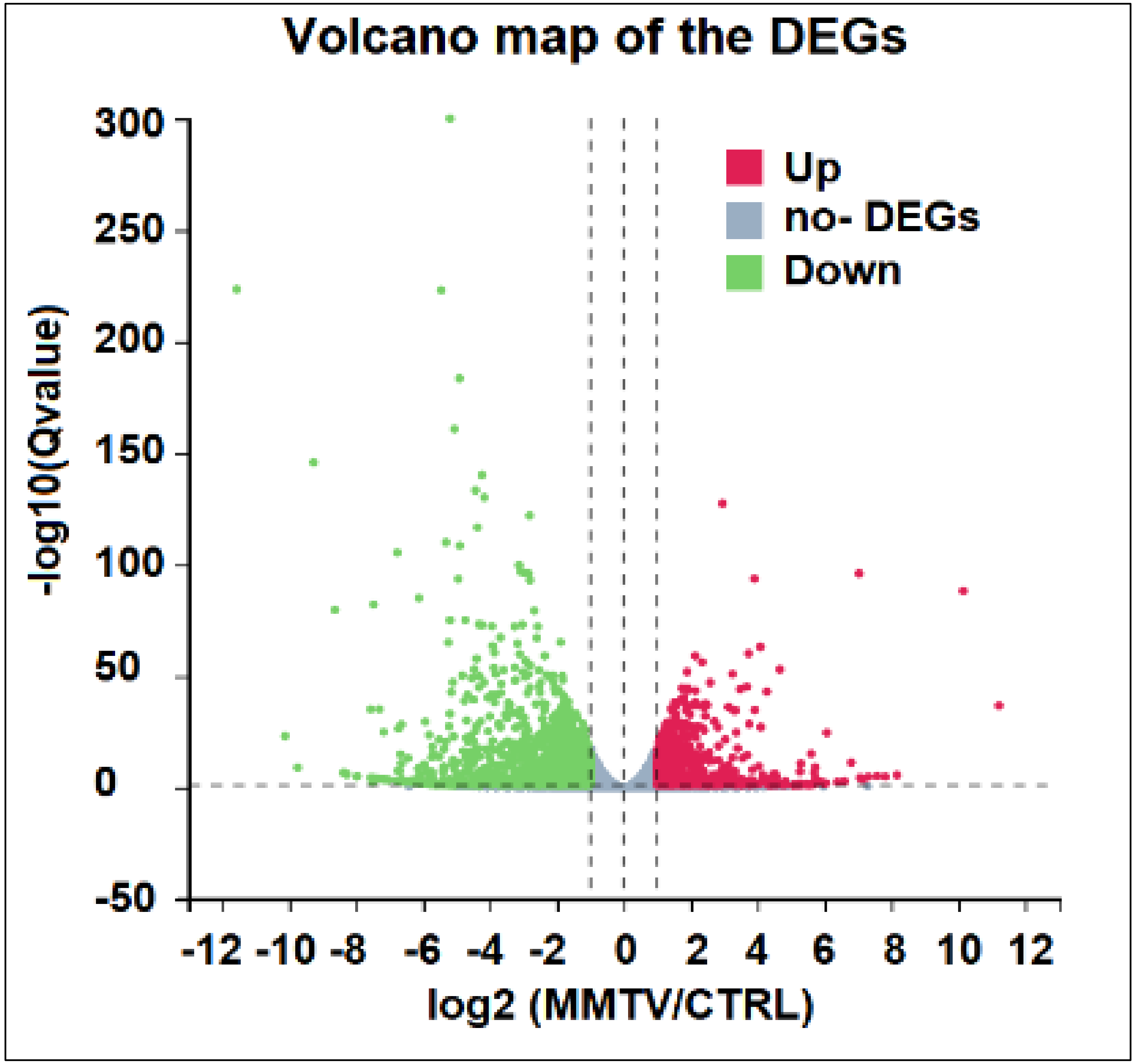

Figure S2: The volcano plot of all DEGs in MMTV/CTRL group. The X- axis represents the fold change of the difference after conversion to log2, and the Y-axis represents the significance value after conversion to log10. Red color represents up regulated DEGs, green represent down regulated DEGs while gray represents no-DEGs.

Figure S3

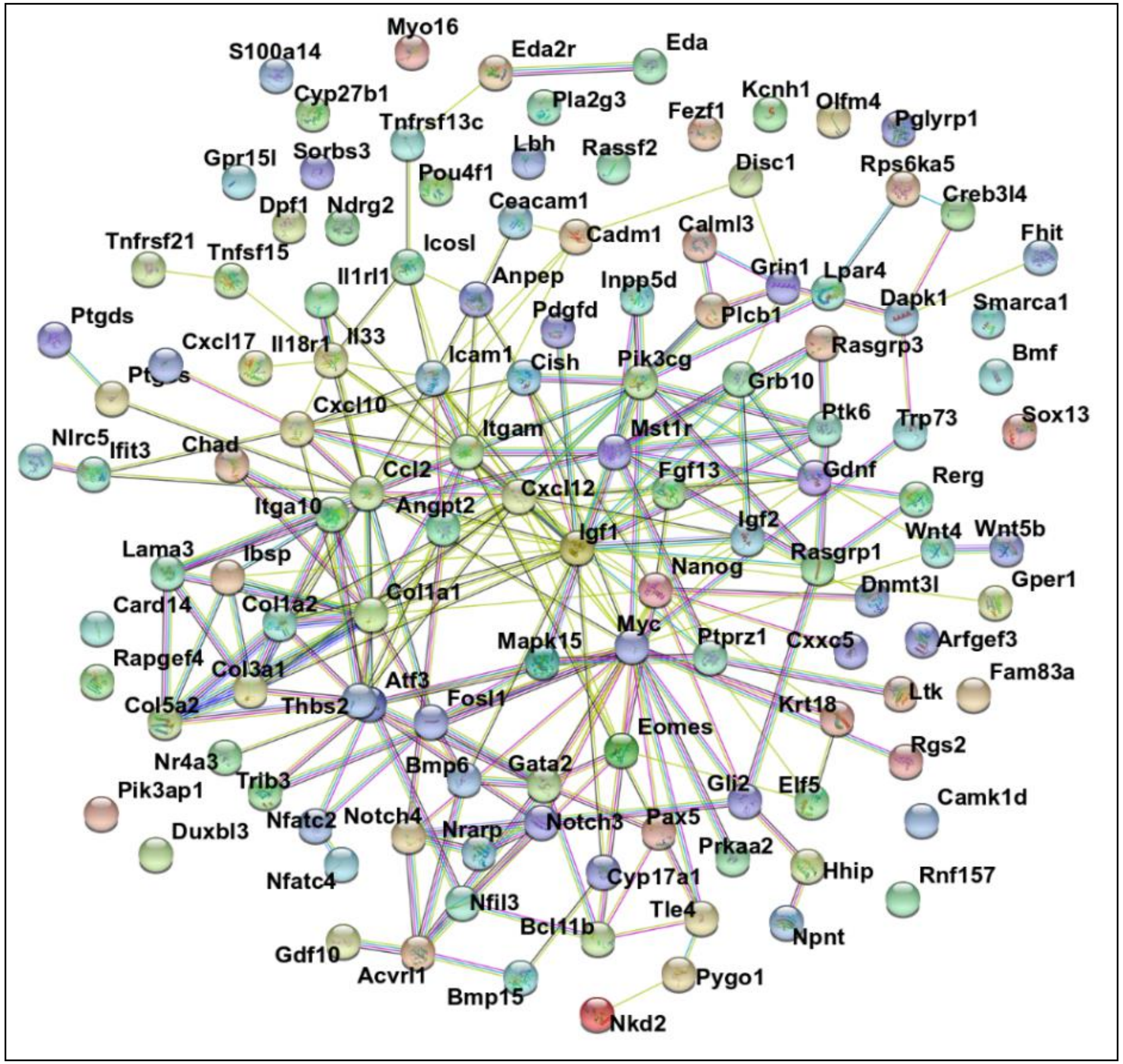

Figure S3: The protein-protein interaction (PPI) network for 122 DEGs present in five or more pathways. STRING was used to generate this network.

Figure S4

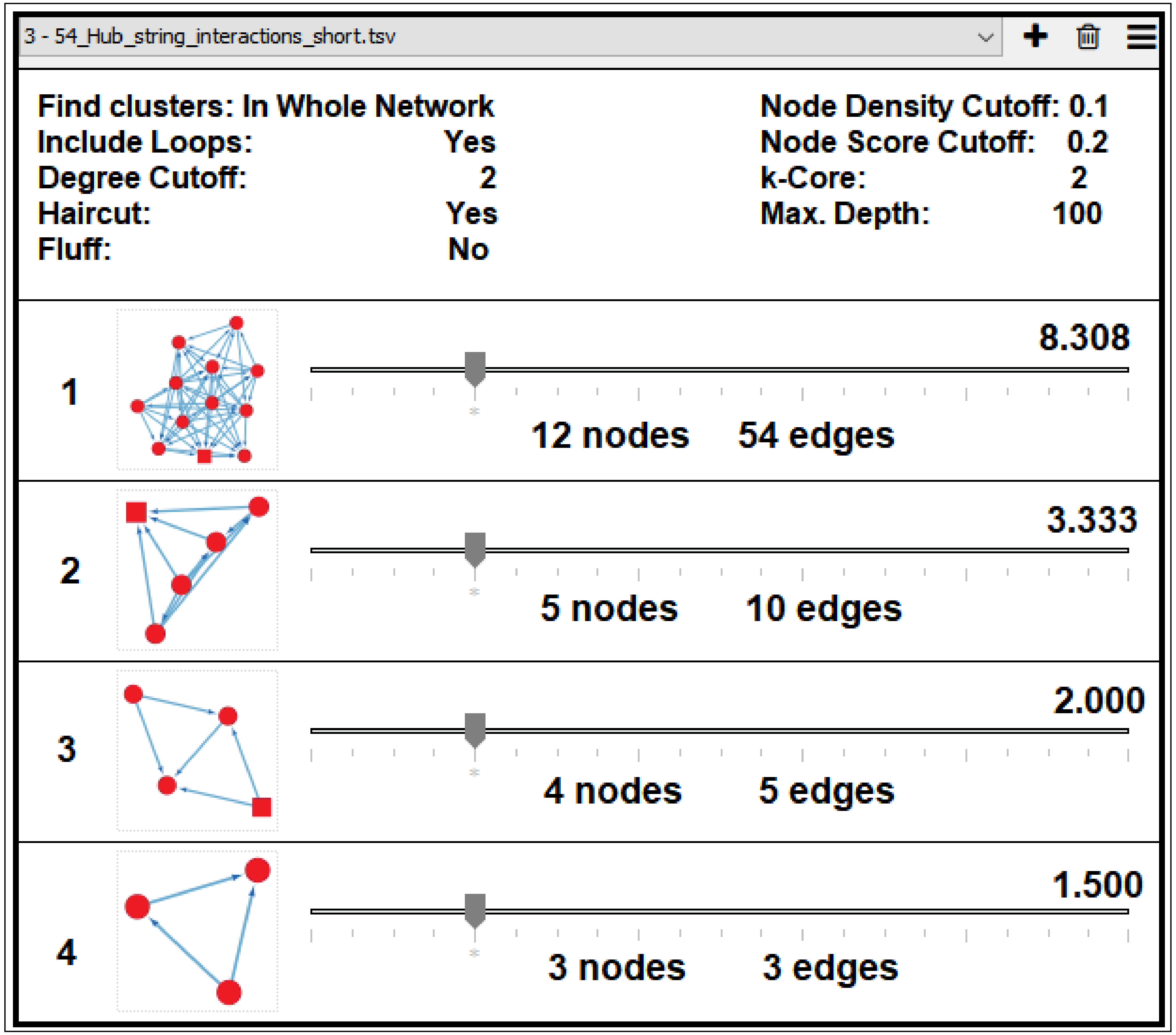

Figure S4: Screenshot of the modules produced after using MCODE. The module with best score was chosen as hub genes.
