# Supplementary material for: Global Down-regulation of Gene Expression Induced by Mouse Mammary Tumor Virus (MMTV) in Normal Mammary Epithelial Cells
Source: Viruses. 2023 May 2;15(5):1110. doi: 10.3390/v15051110 (PMC10223558; doi:10.3390/v15051110)
Supplement: Supplementary file 1 [file viruses-15-01110-s001.zip › Supplementary File S1_Hub Genes.pdf]

Supplementary File S1

Global Downregulation of Gene Expression Induced by Mouse Mammary Tumor Virus (MMTV) in Normal Mammary Epithelial Cells

Waqar Ahmad[1¥], Neena G. Panicker[1¥], Shaima Akhlaq[1], Bushra Gull[1], Jasmin Baby[1], Thanumol A. Khader[1], Tahir A. Rizvi[2,3,4] and Farah Mustafa[1,3*]

[1] Department of Biochemistry and Molecular Biology,
[2] Department of Microbiology and Immunology, CMHS, UAE University, Al Ain, UAE
[3] Zayed Center for Health Sciences (ZCHS), UAE University, Al Ain, UAE
[4] ASPIRE Research Institute in Precision Medicine, Abu Dhabi, UAE University, Al Ain, UAE
[¥] Both authors contributed equally to this work
[*] Correspondence: **Author:** Farah Mustafa, PhD. Department of Biochemistry & Molecular Biology, College of Medicine and Health Sciences (CMHS), UAE University, Tawam Hospital Complex, P.O. Box 15551, Al Ain, UAE Phone: +971-3-713-7509; Fax: +971-3-767-2033; E-mail: fmustafa@uaeu.ac.ae.

Hub Genes and Their Relevance to Viral Infections and MMTV Biology

The upregulated expression of immune regulator chemokine Ccl2 (also called as monocyte chemoattractant Protein-1: MCP-1) is associated with varied immune conditions and clearing acute viral infections, and was observed to be elevated during viral infections [1]. Ccl2 regulates inflammatory monocytes recruitment via its receptor CcR2. Mice deficient in Ccl2 show induced monocyte infiltration [2], whereas, amelioration of CCL2 or CCR2 deficiency could protect against viral infections [3] and associated diseases. Moreover, its overexpression has been observed in cancer/tumor progression and angiogenesis in many cancer types, including human breast cancer [4]. Similarly, upregulation of Ccl2 levels have been associated with disease severity in cancer, especially human breast cancer [5, 6]. Overall, induced expression of Ccl2 in this study might trigger surveillance of cells against MMTV infection, as observed in other viral infections.

We also observed upregulated expression of important immune responsive hub gene Icam1 (Intercellular cell adhesion molecule-1). Icam1 is involved in multi-stage adhesion and transcellular migration. Both epithelial and immune cells show induced Icam1 levels in response to inflammatory stimulation [7]. Icam1 is not only involved in recruiting macrophages and lymphocytes, but there is evidence that it is also used by pathogens, especially viruses, for adhesion and invasion during infection [8]. Icam1 has also been proposed as a possible agent cancer biomarker since it is associated with cancer metastasis [9]. Icam1 activation stimulates TNF and NFƙB pathways that in-turn regulate neutrophil production. The TNF pathway upregulated in our study reflects the innate immune cellular response against MMTV infection in mammary epithelia cells, as detected in bovine mammary epithelial cells and human retinal pigment epithelial cells [10, 11]. Interestingly, Icam1 also triggers NFƙB pathway during the viral infections especially in cold viruses in lung epithelial cells that may enhance cellular inflammatory response and apoptosis to clear the infected cells [12]. We also observed induced NFƙB pathway in our study in the MMTV phenotype, although it was not statistically significant. It has been evident that viral infection dysregulates immune system and increased expression of Icam-1 and other adhesion molecules like integrins and angiogenic proteins [13, 14]. Angiopoietin-2 (Angpt2) gene is a family member of angiopoietin growth factors and is involved in tumorigenesis as invasion, migration and angiogenesis are key factors in cancer cell progression. Moreover, there is also role of angiopoietins in inflammation, cell survival, cell migration and invasion. In our study, MMTV infected cells showed upregulated Angpt2 levels. Induced levels of Angpt2 were observed in patients infected with dengue virus [15] and by other viral infections [16], and are associated with neoplasia. Angpt2 was also shown

to be upregulated in multiple inflammatory diseases and may induce endothelial cell apoptosis by disrupting vascular remodeling of angiopoietin 1 [17-19]. Although tumor angiogenesis is associated with increased Vegf, we found downregulation of Vegf pathway in our study. Interestingly, Vegf positively regulate angiogenesis and many Vegf inhibitors have been used as possible cancer therapies [20].

We observed downregulated expression of Itgam hub gene in MMTV-infected cells. (Fig. 7). Itgam is a member of integrin α subfamily that modulates several diseases like cancer, inflammation, and other infectious diseases. Itgam may be involved in adhesion and tumor cell dispersal in cancers [21]. Integrins performs several normal cellular functions, including attachment to extracellular regions, cell adhesion, cell movement that mediates signal transduction. Thus disruption of Itgam expression could affect apoptosis, proliferation, differentiation, and invasion [22]. Although the precise mechanism of action of Itgam has not been elucidated, it has been observed to be upregulated in normal cells and not in adjacent cancerous cells [23].

Another important hub gene upregulated in our study was an earliest known oncogene Myc (Fig. 7). Myc activation has been known as a hallmark of cancer initiation and progression as its upregulation has been found to induce angiogenesis by suppressing host immune responses [24]. A recent study by Khalid et al found positive correlation between MMTV presence and c-Myc over-expression in invasive breast cancer samples [25]. Interestingly, Myc suppression resulted in restoration of normal cellular activities and finally reversal of tumorigenesis [26, 27]. Upregulated Myc levels induce apoptosis, a safeguard mechanism against the viral infection [28]. We also observed induced apoptotic pathway in MMTV infected cells (Table 2). Myc has been found to regulate multiple cellular pathways, like cell cycle, energy metabolism, and cellular stress response. Interestingly, under normal cellular conditions, Myc activities are regulated under the strict umbrella of several signaling pathways, including Wnt, Notch, Hedgehog and Tgfb [29]. Remarkably, most of these pathways were downregulated after MMTV expression in our study.

We found differential regulation of multiple genes associated with extracellular matrix (ECM). Downregulation of genes expressing most abundant ECM proteins known as collagens was an interesting observation. These genes include Col1a1, Col1a2, Col6a3 and Col9a2 after MMTV expression (Fig. 7). In our study, Col1a1 and Col1a2 were predicted as hub genes that transcribe collagen proteins 1 and 2, respectively, another important component of ECM. Both Col1a1 and Col1a2 have been proposed as diagnostic markers in different cancer types [30, 31] and induced levels of these were observed in most cancers, including human breast cancer, as shown in Fig. 6d. Although the exact role of collagens is not clear during viral infection, MHV68 viral-infected mouse model showed upregulation of lncRNAs that are associated with collagens [32]. It is worth to note that collagens not only support cellular mechanical structure, but also block infective agents, including viruses. Viruses and other infectious agents secrete collagenases and other proteases to destroy collagen barriers [33, 34]. As ECM proteins are involved in regulating cancer cell behavior and tumorigenesis, any irregularity in ECM proteins may alter tissues integrity and structure [35]. For example, expression of CD34 (a hub gene in our study), a cell surface sialomucin, plays an important role in vessel development and function. In fact, several hematopoietic cells containing CD34 can repress tumor growth after their interaction with tumor cells through modulating angiogenesis, tissue remodeling and immune response [36, 37]. However, at the same time, CD34 can induce tumors by maintaining appropriate vessel assembly [38]. Thus, there is a possibility that reduced CD34 expression might facilitate tumor progression after prolonged MMTV expression. Elastin (Eln) is another ECM-associated fibrous protein involved in tissues integrity, and higher levels of Eln protein are associated with many cancers [39]. We hypothesize that reduction in ECM-associated genes expression is a MMTV-induced phenomenon to ease cellular entry.

There was also reduced expression of Acta2 after MMTV expression (Fig. 7). Like ECM genes, Acta2 is also responsible for maintaining cell shape, structure and movement [40]. Acta2 levels were found to be downregulated in most of cancers as well as in breast cancers (Fig. 10). Acta2 downregulation in human lung adenocarcinoma PC14PE6 cells results in impaired migration, invasion, penetration and clonogenicity of diseased cells. This causes lower metastatic potential of cells without changing their tumorigenic potential [41].

Most of the hub genes found in this study were associated with host immune response and most of the chemokines and their receptors are likely to be targeted during pathogen attack. Cxcl12 (Chemokine C-X-C motif chemokine ligand, also known as SDF-1 or stromal cell-derived factor-1) along with its receptor, CxcR4, has been shown to modulate several cellular mechanisms like immune surveillance, hematopoiesis and development [42, 43]. The CxcRr-Cxcl12 axis has been found to be exploited during virus entry. Upregulated levels of CxcR4 were found to facilitate viruses and microbial infections in WHIM by reducing white blood cells and neutrophils [44]. Meanwhile, induced CxcR4 expression was associated with metastasis and inhibition of Cxcl12 was found to reduce metastasis in mouse models by reinforcing its normal functions [44, 45]. It is interesting to note that the CxcRr-Cxcl12 axis has been identified as a main character of viral entry in retroviruses. During HIV infection, virus entry in the host cells is accomplished by forming a complex between env protein gp120 and host CD4 with CxcR4 [46]. Up-regulation of Cxcl12 and cxcR4 was also found during HTLV-1 infection [47]. This expression was mediated through Tax1, an oncoprotein encoded by HTLV-1 [48]. Reduced Cxcl12 levels in our study may be a host response to avoid viral entry in MMTV-infected cells.

Another important target suggested as a hub gene in our study is insulin-like growth factor-1 (Igf1) (Fig. 7). Upon interaction with receptor Igf-1R, two most important cellular pathways, PI3K-AKT and MAPK, get activated that are associated with cell proliferation and anti-apoptosis cascades [49, 50]. It has been evident that most of the viruses utilize PI3-AKT pathway during cell entry and inhibition of PI3-AKT pathways could block viral entry [50]. Most of the viruses either hijack the Igf-1R signaling or interfere with PI3-AKT/ Ras/ MAPK signaling pathways and interfere with phosphorylation of several cellular proteins to ease viral infection and replication [50-54]. Interestingly, activation of Ifg receptor by Igf1 or Igf2 triggers activation of PI3-AKT-mTOR, Ras, Raf, Foxo and Mapk cascades [55]. Thus, downregulation of these pathways observed in our study during post MMTV expression could be due to reduced Igf1 or Igf2 levels implicated in cellular defense system to reduce viral entry and replication.

Meanwhile, MMTV It is secreted in the mother's milk by the mammary epithelial cells and targets dendritic, B- and T-cells in the Peyer's patches found in the gut of suckling pups [56], we found some differentially-regulated genes associated with these cells. These include, Inpp5d, Rasgrp3, Nfatc4, Pik3ap1, Nfatc2 and Pik3cg for B cell- and Nfatc4, Nfatc2, Rasgrp1 and Pik3cg for T cell-associated pathways. These genes are included in the supplementary material sheet S7.

References

1. Deshmane, S. L.; Kremlev, S.; Amini, S.; Sawaya, B. E., Monocyte chemoattractant protein-1 (MCP-1): an overview. *J Interferon Cytokine Res* **2009,** 29, (6), 313-26.
2. Kim, J. H.; Patil, A. M.; Choi, J. Y.; Kim, S. B.; Uyangaa, E.; Hossain, F. M.; Park, S. Y.; Lee, J. H.; Kim, K.; Eo, S. K., CCL2, but not its receptor, is essential to restrict immune privileged central nervous system-invasion of Japanese encephalitis virus via regulating accumulation of CD11b(+) Ly-6C(hi) monocytes. *Immunology* **2016,** 149, (2), 186-203.
3. Lai, C.; Wang, K.; Zhao, Z.; Zhang, L.; Gu, H.; Yang, P.; Wang, X., C-C Motif Chemokine Ligand 2 (CCL2) Mediates Acute Lung Injury Induced by Lethal Influenza H7N9 Virus. *Front Microbiol* **2017,** 8, 587.
4. Hao, Q.; Vadgama, J. V.; Wang, P., CCL2/CCR2 signaling in cancer pathogenesis. *Cell Commun Signal* **2020,** 18, (1), 82.
5. Nagarsheth, N.; Wicha, M. S.; Zou, W., Chemokines in the cancer microenvironment and their relevance in cancer immunotherapy. *Nat Rev Immunol* **2017,** 17, (9), 559-572.
6. Ueno, T.; Toi, M.; Saji, H.; Muta, M.; Bando, H.; Kuroi, K.; Koike, M.; Inadera, H.; Matsushima, K., Significance of macrophage chemoattractant protein-1 in macrophage recruitment, angiogenesis, and survival in human breast cancer. *Clin Cancer Res* **2000,** 6, (8), 3282-9.
7. Bui, T. M.; Wiesolek, H. L.; Sumagin, R., ICAM-1: A master regulator of cellular responses in inflammation, injury resolution, and tumorigenesis. *J Leukoc Biol* **2020,** 108, (3), 787-799.
8. Shukla, S. D.; Shastri, M. D.; Vanka, S. K.; Jha, N. K.; Dureja, H.; Gupta, G.; Chellappan, D. K.; Oliver, B. G.; Dua, K.; Walters, E. H., Targeting intercellular adhesion molecule-1 (ICAM-1) to

reduce rhinovirus-induced acute exacerbations in chronic respiratory diseases. *Inflammopharmacology* **2022,** 30, (3), 725-735.
9. Nagl, L.; Horvath, L.; Pircher, A.; Wolf, D., Tumor Endothelial Cells (TECs) as Potential Immune Directors of the Tumor Microenvironment - New Findings and Future Perspectives. *Front Cell Dev Biol* **2020,** 8, 766.
10. Khan, M. Z.; Khan, A.; Xiao, J.; Ma, J.; Ma, Y.; Chen, T.; Shao, D.; Cao, Z., Overview of Research Development on the Role of NF-kappaB Signaling in Mastitis. *Animals (Basel)* **2020,** 10, (9).
11. Lee, I. T.; Liu, S. W.; Chi, P. L.; Lin, C. C.; Hsiao, L. D.; Yang, C. M., TNF-alpha mediates PKCdelta/JNK1/2/c-Jun-dependent monocyte adhesion via ICAM-1 induction in human retinal pigment epithelial cells. *PLoS One* **2015,** 10, (2), e0117911.
12. Othumpangat, S.; Noti, J. D.; McMillen, C. M.; Beezhold, D. H., ICAM-1 regulates the survival of influenza virus in lung epithelial cells during the early stages of infection. *Virology* **2016,** 487, 85-94.
13. Whalen, M. J.; Doughty, L. A.; Carlos, T. M.; Wisniewski, S. R.; Kochanek, P. M.; Carcillo, J. A., Intercellular adhesion molecule-1 and vascular cell adhesion molecule-1 are increased in the plasma of children with sepsis-induced multiple organ failure. *Crit Care Med* **2000,** 28, (7), 2600-7.
14. Parikh, S. M.; Mammoto, T.; Schultz, A.; Yuan, H. T.; Christiani, D.; Karumanchi, S. A.; Sukhatme, V. P., Excess circulating angiopoietin-2 may contribute to pulmonary vascular leak in sepsis in humans. *PLoS Med* **2006,** 3, (3), e46.
15. Mayetti, M.; Izzah, A. Z.; Jamsari, J.; Darwin, E.; Somasetia, D. H., The Role of Angiopoietin-2 Gene Mutation on Clinical Severity of Dengue Disease in Children. *Open Access Macedonian Journal of Medical Sciences* **2020,** 8, (B), 1110-1115.
16. Yu, X.; Ye, F., Role of Angiopoietins in Development of Cancer and Neoplasia Associated with Viral Infection. *Cells* **2020,** 9, (2).
17. Bader, G. D.; Hogue, C. W., An automated method for finding molecular complexes in large protein interaction networks. *BMC Bioinformatics* **2003,** 4, 2.
18. Hernandez-Bartolome, A.; Lopez-Rodriguez, R.; Borque, M. J.; Gonzalez-Moreno, L.; Real-Martinez, Y.; Garcia-Buey, L.; Moreno-Otero, R.; Sanz-Cameno, P., Angiopoietin-2/angiopoietin-1 as non-invasive biomarker of cirrhosis in chronic hepatitis C. *World J Gastroenterol* **2016,** 22, (44), 9744-9751.
19. Rigamonti, N.; Kadioglu, E.; Keklikoglou, I.; Wyser Rmili, C.; Leow, C. C.; De Palma, M., Role of angiopoietin-2 in adaptive tumor resistance to VEGF signaling blockade. *Cell Rep* **2014,** 8, (3), 696-706.
20. Chung, A. S.; Lee, J.; Ferrara, N., Targeting the tumour vasculature: insights from physiological angiogenesis. *Nat Rev Cancer* **2010,** 10, (7), 505-14.
21. Goodman, S. L.; Picard, M., Integrins as therapeutic targets. *Trends Pharmacol Sci* **2012,** 33, (7), 405-12.
22. Luo, B. H.; Carman, C. V.; Springer, T. A., Structural basis of integrin regulation and signaling. *Annu Rev Immunol* **2007,** 25, 619-47.
23. Gong, Y. Z.; Ruan, G. T.; Liao, X. W.; Wang, X. K.; Liao, C.; Wang, S.; Gao, F., Diagnostic and prognostic values of integrin alpha subfamily mRNA expression in colon adenocarcinoma. *Oncol Rep* **2019,** 42, (3), 923-936.
24. Gabay, M.; Li, Y.; Felsher, D. W., MYC activation is a hallmark of cancer initiation and maintenance. *Cold Spring Harb Perspect Med* **2014,** 4, (6).
25. Khalid, H. F.; Ali, A.; Fawad, N.; Rafique, S.; Ullah, I.; Rehman, G.; Shams, M. U.; Idrees, M., MMTV-LIKE virus and c-myc over-expression are associated with invasive breast cancer. *Infect Genet Evol* **2021,** 91, 104827.
26. Li, Y.; Casey, S. C.; Felsher, D. W., Inactivation of MYC reverses tumorigenesis. *J Intern Med* **2014,** 276, (1), 52-60.

27. Chen, H.; Liu, H.; Qing, G., Targeting oncogenic Myc as a strategy for cancer treatment. *Signal Transduct Target Ther* **2018,** 3, 5.
28. Ahmadi, S. E.; Rahimi, S.; Zarandi, B.; Chegeni, R.; Safa, M., MYC: a multipurpose oncogene with prognostic and therapeutic implications in blood malignancies. *J Hematol Oncol* **2021,** 14, (1), 121.
29. Dang, C. V., MYC on the path to cancer. *Cell* **2012,** 149, (1), 22-35.
30. Hu, Y.; Li, J.; Luo, H.; Song, W.; Yang, J., Differential Expression of COL1A1, COL1A2, COL6A3, and SULF1 as Prognostic Biomarkers in Gastric Cancer. *Int J Gen Med* **2021,** 14, 5835-5843.
31. Li, J.; Ding, Y.; Li, A., Identification of COL1A1 and COL1A2 as candidate prognostic factors in gastric cancer. *World J Surg Oncol* **2016,** 14, (1), 297.
32. Pan, J.; Mor, G.; Ju, W.; Zhong, J.; Luo, X.; Aldo, P. B.; Zhong, M.; Yu, Y.; Jenkins, E. C.; Brown, W. T.; Zhong, N., Viral Infection-Induced Differential Expression of LncRNAs Associated with Collagen in Mouse Placentas and Amniotic Sacs. *Am J Reprod Immunol* **2015,** 74, (3), 237-57.
33. Stavolone, L.; Lionetti, V., Extracellular Matrix in Plants and Animals: Hooks and Locks for Viruses. *Front Microbiol* **2017,** 8, 1760.
34. Makarova, K. S.; Aravind, L.; Koonin, E. V., A novel superfamily of predicted cysteine proteases from eukaryotes, viruses and Chlamydia pneumoniae. *Trends Biochem Sci* **2000,** 25, (2), 50-2.
35. Frantz, C.; Stewart, K. M.; Weaver, V. M., The extracellular matrix at a glance. *J Cell Sci* **2010,** 123, (Pt 24), 4195-200.
36. Fiedler, U.; Christian, S.; Koidl, S.; Kerjaschki, D.; Emmett, M. S.; Bates, D. O.; Christofori, G.; Augustin, H. G., The sialomucin CD34 is a marker of lymphatic endothelial cells in human tumors. *Am J Pathol* **2006,** 168, (3), 1045-53.
37. Sidney, L. E.; Branch, M. J.; Dunphy, S. E.; Dua, H. S.; Hopkinson, A., Concise review: evidence for CD34 as a common marker for diverse progenitors. *Stem Cells* **2014,** 32, (6), 1380-9.
38. Maltby, S.; Freeman, S.; Gold, M. J.; Baker, J. H.; Minchinton, A. I.; Gold, M. R.; Roskelley, C. D.; McNagny, K. M., Opposing roles for CD34 in B16 melanoma tumor growth alter early stage vasculature and late stage immune cell infiltration. *PLoS One* **2011,** 6, (4), e18160.
39. Li, J.; Xu, X.; Jiang, Y.; Hansbro, N. G.; Hansbro, P. M.; Xu, J.; Liu, G., Elastin is a key factor of tumor development in colorectal cancer. *BMC Cancer* **2020,** 20, (1), 217.
40. Pollard, T. D.; Cooper, J. A., Actin, a central player in cell shape and movement. *Science* **2009,** 326, (5957), 1208-12.
41. Lee, H. W.; Park, Y. M.; Lee, S. J.; Cho, H. J.; Kim, D. H.; Lee, J. I.; Kang, M. S.; Seol, H. J.; Shim, Y. M.; Nam, D. H.; Kim, H. H.; Joo, K. M., Alpha-smooth muscle actin (ACTA2) is required for metastatic potential of human lung adenocarcinoma. *Clin Cancer Res* **2013,** 19, (21), 5879-89.
42. Ray, P.; Stacer, A. C.; Fenner, J.; Cavnar, S. P.; Meguiar, K.; Brown, M.; Luker, K. E.; Luker, G. D., CXCL12-gamma in primary tumors drives breast cancer metastasis. *Oncogene* **2015,** 34, (16), 2043-51.
43. O'Bryan, S. M.; Mathis, J. M., CXCL12 Retargeting of an Oncolytic Adenovirus Vector to the Chemokine CXCR4 and CXCR7 Receptors in Breast Cancer. *J Cancer Ther* **2021,** 12, (6), 311-336.
44. Arnolds, K. L.; Spencer, J. V., CXCR4: a virus's best friend? *Infect Genet Evol* **2014,** 25, 146-56.
45. Ok, S.; Kim, S. M.; Kim, C.; Nam, D.; Shim, B. S.; Kim, S. H.; Ahn, K. S.; Choi, S. H.; Ahn, K. S., Emodin inhibits invasion and migration of prostate and lung cancer cells by downregulating the expression of chemokine receptor CXCR4. *Immunopharmacol Immunotoxicol* **2012,** 34, (5), 768-78.

46. Moore, J. P.; Trkola, A.; Dragic, T., Co-receptors for HIV-1 entry. *Curr Opin Immunol* **1997,** 9, (4), 551-62.
47. Arai, M.; Ohashi, T.; Tsukahara, T.; Murakami, T.; Hori, T.; Uchiyama, T.; Yamamoto, N.; Kannagi, M.; Fujii, M., Human T-cell leukemia virus type 1 Tax protein induces the expression of lymphocyte chemoattractant SDF-1/PBSF. *Virology* **1998,** 241, (2), 298-303.
48. Boxus, M.; Twizere, J. C.; Legros, S.; Dewulf, J. F.; Kettmann, R.; Willems, L., The HTLV-1 Tax interactome. *Retrovirology* **2008,** 5, 76.
49. Brahmkhatri, V. P.; Prasanna, C.; Atreya, H. S., Insulin-like growth factor system in cancer: novel targeted therapies. *Biomed Res Int* **2015,** 2015, 538019.
50. Jozefiak, A.; Larska, M.; Pomorska-Mol, M.; Ruszkowski, J. J., The IGF-1 Signaling Pathway in Viral Infections. *Viruses* **2021,** 13, (8).
51. Diehl, N.; Schaal, H., Make yourself at home: viral hijacking of the PI3K/Akt signaling pathway. *Viruses* **2013,** 5, (12), 3192-212.
52. Wojcechowskyj, J. A.; Didigu, C. A.; Lee, J. Y.; Parrish, N. F.; Sinha, R.; Hahn, B. H.; Bushman, F. D.; Jensen, S. T.; Seeholzer, S. H.; Doms, R. W., Quantitative phosphoproteomics reveals extensive cellular reprogramming during HIV-1 entry. *Cell Host Microbe* **2013,** 13, (5), 613-623.
53. Keating, J. A.; Striker, R., Phosphorylation events during viral infections provide potential therapeutic targets. *Rev Med Virol* **2012,** 22, (3), 166-81.
54. Griffiths, C. D.; Bilawchuk, L. M.; McDonough, J. E.; Jamieson, K. C.; Elawar, F.; Cen, Y.; Duan, W.; Lin, C.; Song, H.; Casanova, J. L.; Ogg, S.; Jensen, L. D.; Thienpont, B.; Kumar, A.; Hobman, T. C.; Proud, D.; Moraes, T. J.; Marchant, D. J., IGF1R is an entry receptor for respiratory syncytial virus. *Nature* **2020,** 583, (7817), 615-619.
55. Mirdamadi, Y.; Thielitz, A.; Wiede, A.; Goihl, A.; Papakonstantinou, E.; Hartig, R.; Zouboulis, C. C.; Reinhold, D.; Simeoni, L.; Bommhardt, U.; Quist, S.; Gollnick, H., Insulin and insulin-like growth factor-1 can modulate the phosphoinositide-3-kinase/Akt/FoxO1 pathway in SZ95 sebocytes in vitro. *Mol Cell Endocrinol* **2015,** 415, 32-44.
56. Nandni, S. M., C. M., Mammary Neoplasia in Mice. *Advances in Cancer Research* **1973,** 17, 353-414.
